# Changes in household, transport and recreational physical activity and television viewing time across the transition to retirement: longitudinal evidence from the EPIC-Norfolk cohort

**DOI:** 10.1136/jech-2013-203225

**Published:** 2013-12-03

**Authors:** Inka Barnett, Esther van Sluijs, David Ogilvie, Nicholas J Wareham

**Affiliations:** 1 UKCRC Centre for Diet and Activity Research (CEDAR), University of Cambridge, Cambridge, UK; 2 MRC Epidemiology Unit and UKCRC Centre for Diet and Activity Research (CEDAR), University of Cambridge, Cambridge, UK

**Keywords:** Physical Activity, Health Behaviour, Ageing

## Abstract

**Background:**

Retirement is associated with an increase in recreational physical activity but its impact on other domains of activity (at home, for transport) and sedentary behaviour, such as time spent watching television (TV) is unknown. We examined the association between retirement and changes in domain-specific and overall activity and TV viewing.

**Methods:**

Data were derived from the population-based EPIC (European Prospective Investigation into Cancer)–Norfolk cohort. Physical activity and TV viewing time were self-reported at baseline (1997–2000) and follow-up 2 (2006–2007) by 3334 participants employed at baseline, of whom 785 (24%) were retired at follow-up 1 (2002–2006). Multivariable regression models were fitted to estimate the association between retirement and changes in physical activity and weekly TV viewing time.

**Results:**

Compared with continued employment, retirement was associated with a decline in overall activity (men: non-manual, −40.9 MET h/wk; manual, −49.6 MET h/wk; women: non-manual, −26.9 MET h/wk; manual, −31.6 MET h/wk; all p<0.001 (MET, metabolic equivalent of task)). Domain-specific activity declined for transport and occupational (p<0.001) and increased for recreational (p<0.02) and household (p≤0.002) activity. We observed significant interaction between retirement and social class in respect of overall and domain-specific activity apart from household activity. Retirement was associated with a mean increase in TV viewing time, with the largest increase among manual social classes (men: +3.9 h/wk; women: +2.8 h/wk; both p<0.001).

**Conclusions:**

Interventions should aim to promote household and transport as well as recreational activity. Further research on the impact of retirement on sedentary behaviour is needed.

## Introduction

Physical activity is important for maintaining health, mobility and well-being in older age.^1^ ^2^ Despite these well-established benefits, global physical activity surveillance data show that adults over the age of 60 years are the least active segment of the adult population.^3^ ^4^ Another concern is the high level of sedentary behaviour among older adults^5^ because this is a distinct health behaviour that has been found to be associated with increased cardio-metabolic risk independent of physical activity.^6^ The rapidly aging population, the modest success of existing physical activity interventions,^7^ and the current lack of interventions to reduce sedentary behaviour among older adults^8^ highlight a need to develop effective interventions to promote and maintain an active lifestyle in older age.

The transition to retirement might be an important time period in this respect. Retirement requires individuals to reconsider their established habits and develop new daily routines.^9^ Evidence indicates that older adults might be particularly receptive to changing their behaviours and adopting a healthier lifestyle during this transition period.^10^ A recent systematic review^11^ on physical activity and the transition to retirement suggested an increase in recreational physical activity after retirement, while findings on overall physical activity were inconsistent. There was also some evidence suggesting that the effect of retirement on physical activity varied by sex, with men becoming slightly more physically active than women following retirement. The available evidence was limited by the imprecise assessment of physical activity: many studies relied on single items of unknown validity that are unlikely to have captured all activities of daily living (eg, household chores, active travel) and might have low responsiveness to change. Furthermore, only one study investigated the association between retirement and physical activity specific to a domain other than recreation, namely transport.^12^ The narrow focus on recreational or overall physical activity in the other studies might have missed important avenues for future interventions. In particular, household and transport physical activities have been associated with numerous health benefits among adults in recent studies.^13^ ^14^

A small number of studies^14–17^ have indicated that socioeconomic status might moderate the relationship between retirement and physical activity. Among retirees from lower social classes, recreational and overall physical activity consistently declined, whereas physical activity increased among retirees from higher social classes. It is unknown whether socioeconomic status also moderates the association between retirement and physical activity in other domains. The transition to retirement has also been associated with an increase in sedentary behaviour in two studies.^18^ ^19^ However, additional research is needed to confirm this association.

The aim of this study was to examine the association of retirement with changes in overall and domain-specific (recreational, occupational, household and transport) physical activity and sedentary behaviour as measured by television (TV) viewing time.

## Data and methods

### Study population

**Figure 1:**
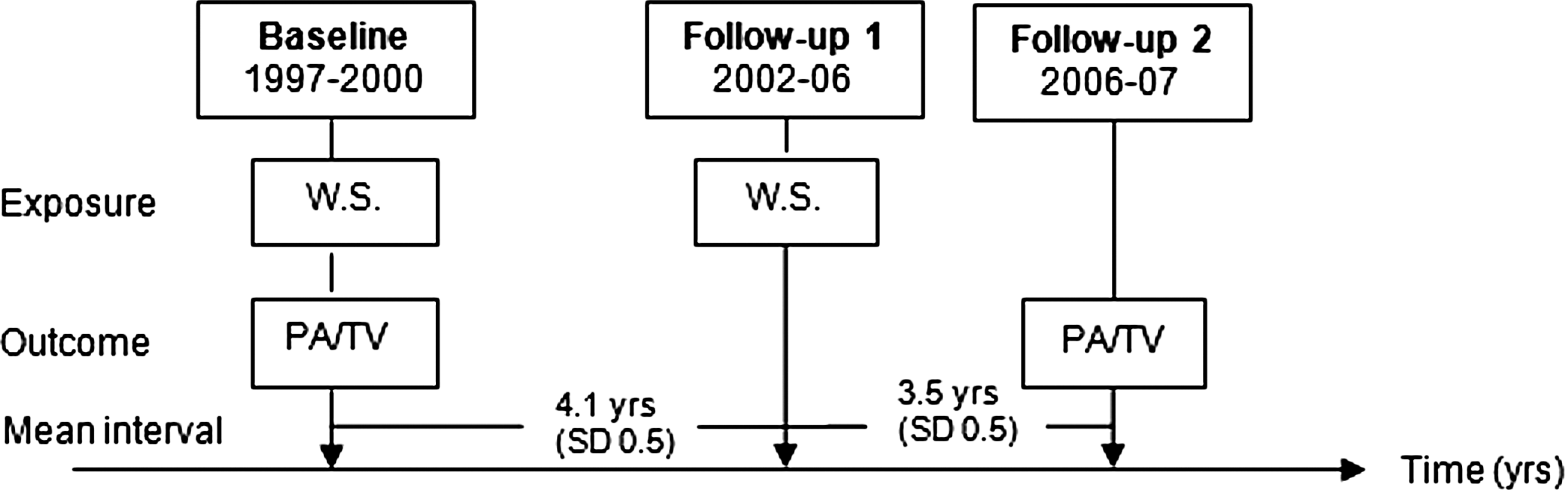
Timeline of data collection for this analysis, European Prospective Investigation into Cancer–Norfolk cohort (W.S., working and retirement status; PA, physical activity (overall and domain-specific); TV, time spent watching television; yrs, years).

Data for this analysis came from the European Prospective Investigation into Cancer and Nutrition (EPIC)–Norfolk study, a population-based cohort study of 25 639 men and women aged 45–79 years at entry and residing in Norfolk, UK. Participants completed a detailed initial survey on their health status and behaviour, and attended a health examination between 1993 and 1997 (Health Check 1). Detailed descriptions of the recruitment, study design and methods have been published elsewhere.^20^ The EPIC–Norfolk study was approved by the Norwich District Health Authority Ethics Committee, and all participants provided written informed consent. The sample for this analysis was drawn from 10 157 participants who responded to Health Check 2 administered between 1997 and 2000 (from here on referred to as ‘baseline’), follow-up 1 between 2002 and 2006 and follow-up 2 between 2006 and 2007 (figure 1). While data on physical activity were also collected in Health Check 1, the methods employed to assess physical activity were different, and resulting data are not comparable with Health Check 2 and the follow-up rounds. Data from Health Check 1 were therefore not included in this analysis.

We restricted our analysis to the subset of participants who were employed at baseline (n=4375) and excluded those who were retired (n=4751) and those who were unemployed, or students, or who described themselves as housewives or househusbands (n=1031). We also excluded participants who had a chronic disease (cancer, stroke, type 2 diabetes or heart disease) that could affect their ability to be physically active (n=239), and participants with missing data for physical activity (occupational physical activity at follow-up 2: n=535; physical activity in other domains and/or rounds: n=92) or covariates (n=29) and those who were made redundant or ceased to work for reasons other than retirement between follow-ups 1 and 2 (n=146), leaving 3334 participants included in analysis.

### Measurement of retirement status

In this study, retirement was defined as the complete withdrawal from work. Realising the multifaceted nature and the increasing complexity of the concept of retirement,^21^ we identified complete retirement based on self-reported retirement status, employment status and occupational physical activity. Retirement and employment status were assessed at baseline and follow-up 1 by asking participants whether they were currently retired from their main occupation and whether they had a paid job at present in each round. Participants who said they were retired but currently had a paid job, or reported occupational physical activity, were classified as employed. Based on the changes in retirement, employment status and occupational physical activity between baseline and follow-up 1, participants were categorised as having ‘remained employed’ or as having made the transition from ‘employed to retired’. Retirement transition status was determined, on average, 3.5 (SD 0.5) years prior to the assessment of follow-up physical activity and TV viewing time (figure 1). This interval enabled us to account for the adjustment to retirement, a period which is a euphoric period full of activities for some retirees, and a period of confusion and inactivity for others.^22^

### Measurement of physical activity and TV viewing time

Physical activity and TV viewing time were assessed at baseline and follow-up 2. Physical activity was self-reported using the comprehensive EPAQ2 physical activity questionnaire (http://www.srl.cam.ac.uk/epic/questionnaires/epaq2/epaq2.pdf).^23^ The validity and repeatability of the EPAQ2 questionnaire has been demonstrated in populations of similar age.^23^ EPAQ2 assesses physical activities over the past year in the context in which they were undertaken and in the following domains: recreational (exercise, sports, walking and cycling for pleasure, do-it-yourself (DIY), gardening), household (activities in and around the house including household chores and caregiving), transport (walking and cycling for non-work and work-related journeys) and occupational physical activity. Overall physical activity was the sum of physical activity in the different domains. Based on the frequency, intensity and duration per episode of physical activity, weekly energy expenditure in each domain was estimated by multiplying the episodes of participation (h/wk) by the metabolic cost (MET) of each activity.^24^ Overall weekly energy expenditure was calculated by summing energy expenditure in all domains. TV viewing time (h/wk) was assessed by summing the responses to four questions about TV viewing before and after 18:00 on weekdays and weekends. Within-subject change in overall and domain-specific physical activity and time spent watching TV was determined by subtracting the values at baseline from the follow-up 2.

### Covariates

Social class was based on the occupation reported in the initial EPIC survey (1993–1997) and categorised into five social classes (with class III subdivided into manual and non-manual class) following the Registrar General's occupation-based classification scheme.^25^ For this study, social classes were collapsed into non-manual social class (consisting of classes I, II, III non-manual), and manual social class (classes III manual, IV, V). Body Mass Index (BMI) was calculated using weight and height measured by trained research assistants at baseline. Based on reported alcohol consumption and smoking status at baseline, participants were categorised as non-drinker or drinker and smoking status as never, former or current. Incidence of a chronic disease was a binary variable assigned according to whether any of the following diseases were diagnosed between baseline and follow-up 1: type 2 diabetes, myocardial infarction, cardiovascular disease or cancer. Change in marital status was based on comparing data from the baseline and follow-up 1, and categorised as remained married, remained single (included widowed or separated), from single to married, or from married to single.

## Analytic methods

Baseline characteristics of the sample, and baseline energy expenditure for physical activity and TV viewing time, were compared between participants who had retired and participants who remained employed at follow-up 1 using t tests for normally distributed continuous variables and χ^2^ tests for categorical variables. Multivariable regression models were fitted to estimate the association between retirement and changes in physical activity and weekly TV viewing time. The reference exposure category comprised those individuals who did not experience the transition to retirement (remained employed). Models were adjusted for age, BMI, smoking, alcohol consumption and physical activity or TV viewing time (as appropriate) at baseline, and for changes in marital status and incidence of chronic disease between baseline and follow-up 1.

Interaction terms for social class and sex were introduced in the regression models, and the significance of improvements in model fit was assessed using likelihood ratio tests. There was a statistically significant interaction between retirement and social class for changes in overall (p<0.006), recreational (p=0.02), transport (p=0.002), and occupational physical activity (p<0.001) and TV viewing time (p=0.04), but not for household physical activity. A significant sex-retirement interaction was found for changes in overall (p<0.001) and occupational physical activity (p<0.001). Analyses were therefore stratified by social class and sex. STATA V.11 was used for the analysis.

## Results

Table 1 presents baseline characteristics of the sample by retirement status at follow-up 1. A total of 370 (23%) men and 415 (24%) women had retired. These participants were older (p<0.001) and had slightly higher BMI (p=0.005) on average than participants who remained in work.

**Table 1:** Descriptive characteristics at baseline (1997–2000) by retirement status at follow-up 1 (2002–2006), EPIC–Norfolk cohort, UK

n=3334	Employed to retired n=785		Remained employed n=2549		p Value
Sex (n, %)					
Male	370	23.1	1230	76.9	0.58
Female	415	23.9	1319	76.1	
Age (mean, SD) years	59.7	4.7	53.0	5.1	<0.001
BMI (mean, SD) kg/m^2^	26.5	3.9	26.0	3.7	0.005
Social class (n, %)					
Non-manual	478	22.6	1637	77.4	0.09
Manual	307	25.2	912	74.8	
Marital status (n, %)					
Married	671	23.2	2226	76.8	0.18
Not married	114	26.1	323	73.9	

BMI, Body Mass Index; EPIC, European Prospective Investigation into Cancer.

Table 2 compares unadjusted baseline physical activity energy expenditure and TV viewing time between participants who had retired at follow-up 1 and participants who remained in work, stratified by social class and sex. Baseline recreational, transport and household physical activity levels were similar among participants who retired and those who continued to work, whereas, occupational physical activity was already significantly lower among participants who were about to retire. Baseline overall physical activity was consistently lower among participants who were about to retire (ie, these participants had retired at follow-up 1), except for women from manual social classes. Women expended most of their weekly energy on activities in and around the home irrespective of their social class, while men expended most energy during work and on recreational activities. Participants who were about to retire watched slightly more TV than people who remained in work.

**Table 2:** Unadjusted overall and domain-specific physical activity and TV viewing time at baseline (1997–2000) stratified by social class and sex, by retirement status at follow-up 1 (2002–2006), EPIC–Norfolk cohort, UK

Baseline PA (MET h/wk)	Non-manual social class						Manual social class					
	Men (n=970)			Women (n=1145)			Men (n=630)			Women (n=589)		
	Mean	SD	p Value	Mean	SD	p Value	Mean	SD	p Value	Mean	SD	p Value
Overall PA												
Retired	128.1	51.2	<0.001	133.8	42.9	<0.001	161.7	63.9	0.043	144.2	50.6	0.39
Employed	145.3	49.3		145.3	43.1		173.8	66.1		148.2	48.0	
Recreational PA												
Retired	36.2	33.1	0.41	26.4	30.9	0.52	26.5	24.9	0.52	25.9	22.6	0.97
Employed	34.4	26.1		36.2	29.3		27.6	24.7		25.9	24.8	
Household PA												
Retired	18.6	15.7	0.52	53.6	23.2	0.45	19.4	15.1	0.08	64.1	30.1	0.057
Employed	19.3	14.2		52.3	24.5		17.2	14.3		59.1	26.6	
Transport PA												
Retired	8.3	15.6	0.10	5.6	9.1	0.050	10.1	18.6	0.20	8.4	13.9	0.14
Employed	10.3	15.5		7.0	10.2		8.3	14.2		6.8	9.9	
Occupational PA												
Retired	64.9	36.8	<0.001	47.9	29.7	<0.001	96.4	54.2	0.002	45.9	32.8	<0.001
Employed	81.6	39.8		58.3	31.6		112.5	56.9		56.5	34.0	
TV viewing time (h/wk)												
Retired	17.9	8.9	0.015	19.2	9.0	<0.001	21.8	8.6	0.004	22.3	9.1	0.003
Employed	16.4	7.9		16.6	8.5		19.6	8.2		19.9	8.5	

EPIC, European Prospective Investigation into Cancer; MET, metabolic equivalent of task; PA, physical activity; TV, television.

Tables 3 and 4 present observed mean changes in physical activity and TV viewing time from baseline to follow-up 2, and the results of unadjusted and adjusted multivariable regression models examining the association between retirement and changes in physical activity and TV viewing time. From baseline to follow-up 2, a mean decrease in overall, transport and occupational physical activity was observed in all subgroups. The observed decline was larger among retirees than among participants who remained employed, and greater in retirees from manual social classes, except for occupational physical activity among women. Mean recreational and household physical activity and mean TV viewing time increased during the follow-up period, with consistently higher increases observed among participants who retired.

**Table 3:** Mean changes and associations between retirement and changes in physical activity and TV viewing among men from non-manual and manual social classes, EPIC–Norfolk cohort, UK, 1997–2007

PA (MET h/wk)	Retired n=207		Employed n=763		Unadjusted net effect of retirement			Adjusted net effect of retirement		
	Mean change	SD	Mean change	SD	β	(95% CI)	p Value	β	(95% CI)	p Value
Non-manual social class										
Overall PA	−49.2	53.6	−7.9	52.3	−41.2	(−49.3 to −33.1)	<0.001	−40.9	(−49.5 to −32.5)	<0.001
Recreational PA	13.4	41.6	7.3	33.4	6.1	(0.7 to 11.5)	0.028	7.2	(1.3 to 13.1)	0.017
Household PA	10.6	17.9	4.6	15.3	5.9	(3.5 to 8.4)	<0.001	4.3	(1.6 to 6.9)	0.002
Transport PA	−7.3	15.5	−6.4	13.9	−0.9	(−3.2 to 1.2)	0.39	−2.7	(−3.7 to −1.7)	<0.001
Occupational PA	−64.9	36.8	−12.9	40.6	−52.0	(−58.1 to −45.9)	<0.001	−51.3	(−57.1 to −45.5)	<0.001
TV viewing time (h/wk)	5.4	8.3	2.4	6.4	3.0	(1.9 to 4.1)	<0.001	2.6	(1.4 to 3.8)	<0.001
Manual social class										
Overall PA	−70.5	66.7	−10.0	77.4	−60.5	(−73.9 to −47.2)	<0.001	−49.6	(−63.2 to −35.9)	<0.001
Recreational PA	20.9	42.2	2.2	36.2	18.7	(11.9 to 25.5)	<0.001	17.8	(9.8 to 25.7)	<0.001
Household PA	14.4	23.5	3.7	15.9	10.7	(7.5 to 13.9)	<0.001	11.8	(8.0 to 15.6)	<0.001
Transport PA	−9.1	18.6	−4.6	12.4	−4.4	(−6.9 to −1.9)	0.001	−2.0	(−3.2 to −0.8)	0.001
Occupational PA	−96.4	54.2	−10.7	68.5	−85.7	(−97.3 to −74.0)	<0.001	−78.2	(−88.9 to −67.5)	<0.001
TV viewing time (h/wk)	6.6	8.7	2.3	8.0	4.3	(2.8 to 5.8)	<0.001	3.9	(2.2 to 5.6)	<0.001

Adjusted for age, BMI (Body Mass Index), change in marital status, PA or TV viewing time at baseline, incidence of chronic disease, alcohol consumption, smoking.

EPIC, European Prospective Investigation into Cancer; MET, metabolic equivalent of task; PA, physical activity; TV, television.

**Table 4:** Mean changes and associations between retirement and changes in physical activity and TV viewing among women from non-manual and manual social classes, EPIC–Norfolk Cohort, UK, 1997–2007

	Retired n=271		Employed n=874		Unadjusted net effect of retirement			Adjusted net effect of retirement		
PA (MET h/wk)	Mean change	SD	Mean change	SD	β	(95% CI)	p Value	β	(95% CI)	p Value
Non-manual social class										
Overall PA	−32.8	51.8	−6.3	52.0	−26.5	(−33.6 to −19.4)	<0.001	−26.9	(−33.9 to −19.9)	<0.001
Recreational PA	11.8	34.7	4.8	29.7	6.9	(2.8 to 11.2)	0.001	6.8	(2.4 to 11.1)	0.002
Household PA	9.1	29.3	1.9	25.8	7.1	(3.5 to 10.8)	<0.001	5.1	(1.5 to 8.7)	0.005
Transport PA	−4.6	9.1	−3.9	9.8.	−0.6	(−1.9 to 0.7)	0.35	−1.4	(−2.1 to −0.7)	<0.001
Occupational PA	−47.9	29.7	−8.1	39.4	−39.8	(−44.9 to −34.7)	<0.001	−38.2	(−42.7 to −33.6)	<0.001
TV viewing time (h/wk)	5.3	7.3	2.7	6.9	2.6	(1.6 to 3.5)	<0.001	2.3	(1.3 to 3.3)	<0.001
Manual social class										
Overall PA	−39.3	56.3	−2.6	62.0	−36.7	(−48.1 to −25.2)	<0.001	−31.6	(−43.4 to −19.7)	<0.001
Recreational PA	10.6	31.9	4.9	32.9	5.6	(−0.5 to 11.8)	0.07	4.6	(−2.2 to 11.4)	0.19
Household PA	4.2	34.7	0.5	28.8	4.2	(−1.5 to 9.9)	0.15	5.2	(−0.6 to 11.0)	0.08
Transport PA	−7.4	13.7	−3.1	9.5	−4.2	(−6.2 to −2.2)	<0.001	−2.2	(−3.2 to −1.1)	<0.001
Occupational PA	−45.9	32.8	−3.6	47.8	−42.3	(−50.7 to −33.9)	<0.001	−39.9	(−47.6 to −32.2)	<0.001
TV viewing time (h/wk)	6.6	9.0	2.8	7.7	3.8	(2.3 to 5.4)	<0.001	2.8	(1.2 to 4.5)	0.001

Adjusted for age, BMI (Body Mass Index), change in marital status, PA or TV viewing time at baseline, incidence of chronic disease, alcohol consumption, smoking.

EPIC, European Prospective Investigation into Cancer; MET, metabolic equivalent of task; PA, physical activity; TV, television.

After adjustment for confounders, retirement remained significantly associated with a greater mean decrease in overall physical activity over time compared to staying in employment (men: non-manual, −40.9 MET h/wk; manual, −49.6 MET h/wk; women: non-manual, −26.9 MET h/wk; manual, −31.6 MET h/wk; all p<0.001). Retirement was also associated with a decrease in transport and occupational physical activity among men and women from all social classes; and with a larger mean increase in recreational and household physical activity among men from manual and non-manual social classes, and women from non-manual social classes. No association between retirement and changes in mean recreational and household physical activity could be found for women from manual social classes. Retirement was also associated with a greater mean increase in weekly TV viewing time: the net increase was largest among men and women from manual social classes (men: +3.9 h/wk; women: +2.8 h/wk; both p<0.001).

## Discussion

This large population-based study found that the transition to retirement was consistently associated with a net decline in transport and occupational physical activity, and a net increase in recreational and household physical activity, except for women from manual social classes. The transition to retirement was also associated with a net decrease in overall physical activity. This study found a net increase in TV viewing time following retirement, with participants from manual social classes showing the largest net increase.

A decrease in overall physical activity in retirement has been reported in several previous studies.^15^ ^16^ However, overall physical activity was assessed more comprehensively in this study than in previous studies, permitting us to estimate changes across the retirement transition more accurately. The net decline in overall physical activity was larger among retirees from manual than from non-manual groups. This finding is consistent with those of previous studies,^15–17^ and might be explained by the loss of higher levels of occupational physical activity among retirees from manual occupations (as suggested by higher levels of occupational physical activity at baseline), which is not compensated for by increases in other domains of activity.

The net decline in transport physical activity in retirement might be explained by the loss of work-related active transport after exit from the labour force that was reported in a previous study.^12^ Given that walking (for transport and for recreation) has been shown to be a preferred physical activity among older adults,^26^ the promotion of utilitarian and recreational walking might be an effective approach for maintaining overall physical activity levels after the transition to retirement.

The positive association between retirement and recreational physical activity is supported by the findings from previous studies.^15^ ^27–29^ However, by contrast with those studies, the association observed here was not consistent in that no association was found for women from manual social classes. One explanation might lie in the different assessments of recreational physical activity used in the studies. While this study used self-reports of duration, frequency and intensity of a clearly defined list of exercises, sports and gardening, most previous studies relied on participants’ own concepts of recreational physical activity without providing exact definitions. This might have led to an overestimation of change in recreational physical activities because, as suggested in numerous qualitative studies,^30–32^ older adults have broad understandings of recreational physical activity that may also include household and transport physical activities.

Retirement was associated with a net increase in household physical activity. The influence of retirement on household physical activity has been overlooked in previous studies, but the baseline measures of household physical activity suggest that it might form an important component of overall physical activity in this period of life, particularly for women. Several recent studies have suggested that elderly women in particular, expend most of their total energy on household physical activity, and that exercise and sports might play only a minor role.^33^ ^34^ While household chores and other activities are often of relatively low intensity, they have, nevertheless, been shown to be associated with health benefits in older adults.^35^ Interventions should raise awareness of the potential health benefits of household physical activities and strengthen participants’ ability to perform chores and other activities in and around the home.

Some differences could be observed in the magnitude of the mean changes in physical activity and TV viewing time between occupational social classes. The observed mean decline in overall, transport and occupational physical activity was larger among retirees from manual social classes than among those from non-manual classes. The greater decline is likely to be attributable to the loss of higher levels of occupational physical activity among manual workers compared to non-manual workers. The observed mean increase in recreational physical activity (only for men) and time spent watching TV was larger in manual compared to non-manual workers following retirement. Previous studies have suggested that individuals with manual and physically demanding occupations often engage in less recreational physical activity after working hours and on weekends compared with employees in non-manual occupations.^36^ ^37^ In retirement, occupational physical demands are removed and former manual workers may be more likely to adopt or increase recreational physical activity.

A positive association between retirement and TV viewing time was also reported in one previous study.^29^ Television watching is the most prevalent sedentary behaviour among older adults,^38^ and is an independent predictor for all-cause and cardiovascular mortality and the metabolic syndrome.^39^ ^40^ Further research is needed to examine whether and how an increase in time spent watching TV in retirement is related to the changes in physical activity in other domains of life. Future studies should also investigate the impact of retirement on other sedentary behaviours (eg, doing handicrafts or reading) and examine the independent effect of sedentary behaviour patterns on health in retirement.

One of the main strengths of this study is the detailed and comprehensive assessment of physical activity that permitted the investigation of the association between retirement and domain-specific physical activity. While it is not possible to draw conclusions regarding the absolute levels of physical activity after retirement, to our knowledge this is the first study that has quantified the influence of retirement on changes in the energy expended in different domains of physical activity, thereby providing a more precise assessment of the impact of retirement. Other strengths include the comprehensive assessment of retirement based on three independent measurements, the prospective, population-based study design and the large sample size of the EPIC–Norfolk cohort from which the study sample was drawn.

The study has several limitations. Physical activity and TV viewing time were self-reported and may, therefore, have been affected by measurement bias, such as recall and social desirability bias. For example, the EPAQ2 questionnaire assesses occupational physical activity based on the average number of hours per week spent sitting (doing light or moderate work), standing (doing light or moderate work), walking (carrying nothing or something light, or carrying something heavy) and pushing or moving heavy objects, and the average number of stairs climbed. While this method considers different physical activities employees might engage in during a typical work day, it might not be sensitive enough to detect the variability in the intensity and duration of different occupational physical activities from day to day, especially among manual workers. This may result in an overestimation of occupational physical activity among manual workers and, therefore, of the impact of retirement on their overall physical activity. The transferability of the results might have been limited by the number of observations that were dropped because of missing data on occupational physical activity in follow-up 2. However, results from a sensitivity analysis in which participants with missing occupational physical activity were categorised as retired were consistent with findings from the multivariable regression models (data not shown), suggesting that the effects of the missing observations were negligible. No details on the circumstances or the pathway to retirement that might have affected physical activity or TV viewing behaviours were available. However, participants with a chronic disease at baseline were excluded and analyses were adjusted for the incidence of a chronic disease between baseline and follow-up 1 to minimise the possible effects of ill health on the decision to retire and on physical activity.

In conclusion, this study suggests that the transition to retirement is associated with a net decline in overall physical activity. Retirement might therefore be an important period for promotion of physical activity. Given the limited effectiveness of existing interventions to promote adoption and, in particular, long-term maintenance of physical activity by older adults,^7^ physical activity intervention target at this time period may encourage more sustainable changes in physical activity behaviour. Promoting household and transport physical activity, in addition to recreational physical activity, might be acceptable and appropriate approaches to raising and maintaining overall physical activity in retirement. This study also highlights the need for further research on the impact of retirement on sedentary behaviour patterns.

**What is already known on this subject**

- Retirement has been associated with changes in recreational and overall physical activity.
- The existing evidence is limited by the imprecise measurement of physical activity.
- Evidence on the influence of retirement on physical activity in other domains of daily living (at home, for transport) and on sedentary behaviours is lacking.

**What this study adds**

- Using a comprehensive measure of physical activity, this study finds a net decline in occupational and transport physical activity, and a net increase in household and recreational physical activity following retirement. There was also a net decline in overall physical activity following retirement.
- Promoting household and transport physical activity in addition to recreational physical activity may help support an active lifestyle in retirement.
- Retirement was associated with a net increase in time spent watching TV, highlighting the need for more research on sedentary behaviour in this age group.

**Policy implications**

- To promote healthy and active aging in retirement, a range of physical activities including recreational and household activities and active travel should be encouraged.

## References

[1] Chodzko-Zajko WJ Proctor DN Fiatarone Singh MA Exercise and physical activity for older adults. Med Sci Sports Exerc 2009;41:1510–30 19516148 10.1249/MSS.0b013e3181a0c95c

[2] Nelson ME Rejeski W Blair SN Physical activity and public health in older adults: recommendation from the American College of Sports Medicine and the American Heart Association. Med Sci Sports Exerc 2007;39:1435–45 17762378 10.1249/mss.0b013e3180616aa2

[3] Craig R Mindell J Hirani V Health survey for England 2008: physical activity and fitness. Leeds: National Centre for Social Research with permission of The NHS Information Centre, 2009

[4] Troiano RP Berrigan D Dodd KW Physical activity in the United States measured by accelerometer. Med Sci Sports Exerc 2008;40:181–8 18091006 10.1249/mss.0b013e31815a51b3

[5] Evenson KR Buchner DM Morland KB Objective measurement of physical activity and sedentary behavior among US adults aged 60 years or older. Prev Chronic Dis 2012;9:1–19 PMC3277387 22172193

[6] Owen N Healy GN Matthews CE Too much sitting: the population health science of sedentary behavior. Exerc Sport Sci Rev 2010;38:105–13 20577058 10.1097/JES.0b013e3181e373a2 PMC3404815

[7] Taylor A Cable N Faulkner G Physical activity and older adults: a review of health benefits and the effectiveness of interventions. J Sports Sci 2004;22:703–25 15370483 10.1080/02640410410001712421

[8] Gardiner PA Eakin EG Healy GN Feasibility of reducing older adults’ sedentary time. Am J Prev Med 2011;41:174–7 21767725 10.1016/j.amepre.2011.03.020

[9] Jonsson H Josephsson S Kielhofner G Narratives and experience in an occupational transition: a longitudinal study of the retirement process. Am J Occup Ther 2001;55:424–32 11723987 10.5014/ajot.55.4.424

[10] Lang IA Rice NE Wallace RB Smoking cessation and transition into retirement: analyses from the English Longitudinal study of ageing. Age Ageing 2007;36:638–43 17906305 10.1093/ageing/afm119

[11] Barnett I Sluijs van EMF Ogilvie D Physical activity and transitioning to retirement: a systematic review. Am J Prev Med 2012;43:329–36 22898127 10.1016/j.amepre.2012.05.026 PMC3830178

[12] Slingerland AS Van Lenthe FJ Jukema JW Aging, retirement, and changes in physical activity: prospective cohort findings from the GLOBE study. Am J Epidemiol 2007;165:1356–63 17420180 10.1093/aje/kwm053

[13] Samitz G Egger M Zwahlen M Domains of physical activity and all-cause mortality: systematic review and dose–response meta-analysis of cohort studies. Int J Epidemiol 2011;40:1382–400 22039197 10.1093/ije/dyr112

[14] Wanner M Gaķtschi T Martin-Diener E Active transport, physical activity, and body weight in adults: a systematic review. Am J Prev Med 2012;42:493–502 22516490 10.1016/j.amepre.2012.01.030

[15] Berger U Der G Hannah MK The impact of retirement on physical activity. Ageing Soc 2005;25:181–95

[16] Chung S Domino ME Stearns SC Retirement and physical activity: analyses by occupation and wealth. Am J Prev Med 2009;36:422–8 19269129 10.1016/j.amepre.2009.01.026

[17] Mein GK Shipley MJ Hillsdon M Work, retirement and physical activity: cross-sectional analyses from the Whitehall II study. Eur J Public Health 2005;15:317–22 15941745 10.1093/eurpub/cki087

[18] Evenson KR Rosamond WD Cai J Influence of retirement on leisure-time physical activity. Am J Epidemiol 2002;155:692–9 11943686 10.1093/aje/155.8.692

[19] Touvier M Bertrais S Charreire H Changes in leisure-time physical activity and sedentary behaviour at retirement: a prospective study in middle-aged French subjects. Int J Behav Nutr Phys Act 2010;7:14–23 20181088 10.1186/1479-5868-7-14 PMC2834610

[20] Day N Oakes S Luben R EPIC-Norfolk: study design and characteristics of the cohort. European prospective investigation of cancer. Br J Cancer 1999;80:95–103 10466767

[21] Denton FT Spencer BG What is retirement? A review and assessment of alternative concepts and measures. Can J Aging 2009;28:63–76 19860967 10.1017/S0714980809090047

[22] Atchley RC Retirement: Leaving the world of work. Ann Am Acad Pol Soc Sci 1982:120–31

[23] Wareham NJ Jakes RW Rennie KL Validity and repeatability of the EPIC-Norfolk physical activity questionnaire. Int J Epidemiol 2002;31:168–74 11914316 10.1093/ije/31.1.168

[24] Ainsworth BE Haskell WL Whitt MC Compendium of physical activities: an update of activity codes and MET intensities. Med Sci Sports Exerc 2000;32:498–516 10.1097/00005768-200009001-00009 10993420

[25] Shohaimi S Luben R Wareham N Residential area deprivation predicts smoking habit independently of individual educational level and occupational social class. A cross sectional study in the Norfolk cohort of the European Investigation into Cancer (EPIC-Norfolk). J Epidemiol Community Health 2003;57:270–6 12646543 10.1136/jech.57.4.270 PMC1732421

[26] Eyler AA Brownson RC Bacak SJ The epidemiology of walking for physical activity in the United States. Med Sci Sports Exerc 2003;35:1529–36 12972873 10.1249/01.MSS.0000084622.39122.0C

[27] Lahti J Laaksonen M Lahelma E Changes in leisure-time physical activity after transition to retirement: a follow-up study. Int J Behav Nutr Phys Act 2011;8:1–36 21513555 10.1186/1479-5868-8-36 PMC3094268

[28] Sjoesten N Kivimaeki M Singh-Manoux A Change in physical activity and weight in relation to retirement: the French GAZEL Cohort Study. BMJ open 2011;2:1–13 10.1136/bmjopen-2011-000522 PMC3277904 22318663

[29] Touvier M Bertrais S Charreire H Changes in leisure-time physical activity and sedentary behaviour at retirement: a prospective study in middle-aged French subjects. Int J Behav Nutr Phys Act 2010;7:1–14 20181088 10.1186/1479-5868-7-14 PMC2834610

[30] Beck F Gillison F Standage M A theoretical investigation of the development of physical activity habits in retirement. Br J Health Psychol 2010;15:663–79 19922724 10.1348/135910709X479096

[31] Scanlon-Mogel JM Roberto KA Older adults’ beliefs about physical activity and exercise: life course influences and transitions. Qual Ageing 2004;5:33–44

[32] Witcher CS Holt NL Spence JC A case study of physical activity among older adults in rural Newfoundland, Canada. J Aging Phys Act 2007;15:166–83 17556783 10.1123/japa.15.2.166

[33] Plonczynski DJ Wilbur JE Larson JL Lifestyle physical activity of older rural women. Res Nurs Health 2008;31:501–13 18384050 10.1002/nur.20282 PMC2574920

[34] Ratzlaff CR Good news, bad news: sports matter but occupational and household activity really matter [editorial]. Br J Sports Med 2012;3:1 10.1136/bjsports-2011-090800 22411766

[35] Buman MP Hekler EB Haskell WL Objective light-intensity physical activity associations with rated health in older adults. Am J Epidemiol 2010;172:1155–65 20843864 10.1093/aje/kwq249 PMC3004766

[36] Kirk MA Rhodes RE Occupation correlates of adults’ participation in Leisure-time physical activity: a systematic review. Am J Prev Med 2011;40:476–85 21406284 10.1016/j.amepre.2010.12.015

[37] Van Domelen DR Koster A Caserotti P Employment and physical activity in the US. Am J Prev Med 2011;41:136–45 21767720 10.1016/j.amepre.2011.03.019 PMC5221416

[38] Nielsen Media Research Nielsen Report on Television. In: Research NM, ed. New York, NY: Nielsen Media Research, 2000

[39] Edwardson CL Gorely T Davies MJ Association of Sedentary Behaviour with Metabolic Syndrome: A Meta-Analysis. PLoS ONE 2012;7:1–5 10.1371/journal.pone.0034916 PMC3325927 22514690

[40] Matthews CE George SM Moore SC Amount of time spent in sedentary behaviors and cause-specific mortality in US adults. Am J Clin Nutr 2012;95:437–45 22218159 10.3945/ajcn.111.019620 PMC3260070

